# Effect of Season on Testicular Development and Spermatogenesis in Hu Sheep: Insights from Antioxidant Indices, Oxylipins, and Transcriptomics

**DOI:** 10.3390/ani15192824

**Published:** 2025-09-27

**Authors:** Wanhong Li, Xinyue Zhang, Jie Shen, Xiuxiu Weng

**Affiliations:** State Key Laboratory of Herbage Improvement and Grassland Agro-Ecosystems, College of Pastoral Agriculture Science and Technology, Lanzhou University, Lanzhou 730020, China; zhangxy21@lzu.edu.cn (X.Z.); shenj2023@lzu.edu.cn (J.S.); wengxx@lzu.edu.cn (X.W.)

**Keywords:** antioxidant, Hu sheep, spermatogenesis, oxylipins, testis, transcriptomic

## Abstract

**Simple Summary:**

Numerous studies have investigated the influence of season on spermatogenesis in adult rams. Heat stress during summer exerts adverse effects on spermatogenesis and semen quality. The pre-sexual maturity stage is the most important period for testis development in rams. However, little is known about the effects of seasonal factors on testicular development during the pre-sexual maturity in Hu rams. Therefore, this study investigated the sperm density, histology, antioxidant properties, oxylipins and differentially expressed genes in testicular tissues between winter-born and summer-born Hu rams at six months old. Compared with the winter-born group, the testicular tissues of summer-born rams showed stronger testicular antioxidant capacity and lower lipid peroxidation at sexual maturity stage, contributing to enhanced spermatogenesis.

**Abstract:**

Numerous factors, including genetic, environmental, and nutritional, are involved in testicular development and spermatogenesis. However, little is known about the effects of seasonal factors on pre-sexual maturity testicular development in Hu rams, which are famous for their high fertility and year-round estrus onset. This study explored the effect of the birth season on testicular development and spermatogenesis in Hu sheep. Thirty-six 6-month-old male lambs born in summer (n = 18) and winter (n = 18) were selected for analysis. Results showed that summer-born lambs exhibited significantly higher cauda sperm density (102.65 ± 9.56 vs. 16.86 ± 2.02 × 10^7^/g), antioxidant indices such as superoxide dismutase (SOD: 6.29 ± 1.01 vs. 4.09 ± 0.25 U/mgprot), and higher expression levels of glutathione peroxidase 3 (*GPX3*), glutathione peroxidase 4 (*GPX4*), and copper/zinc superoxide dismutase (*Cu*/*Zn-SOD*) than winter-born lambs. Conversely, the malondialdehyde content (1.08 ± 0.32 vs. 2.13 ± 0.34 nmol/mgprot) was significantly lower in the summer-born group (*p* < 0.05) than in the winter-born group. A total of 44 differential oxylipins and 326 differentially expressed genes (DEGs) were screened by ultra-performance liquid chromatography–tandem mass spectrometry and transcriptomics, respectively. An integrated analysis of oxylipins and transcriptomics revealed that these differential molecules were enriched in metabolic pathways. Notably, downregulated DEGs (e.g., UAP1L1 and NAT8L) were significantly correlated with upregulated differential oxylipins (e.g., epoxyeicosatrienoic acids and dihydroxyeicosatrienoic acids). These results indicate that compared to the winter-born group, the testicular tissues of summer-born rams showed stronger testicular antioxidant capacity and lower lipid peroxidation at the sexual maturity stage, which contributes to spermatogenesis.

## 1. Introduction

A large amount of reactive oxygen species (ROS) is produced during testicular development and spermatogenesis. Those ROS are necessary for maintaining normal physiological functions. Under physiological conditions, the body’s antioxidant system eliminates excess ROS to maintain a redox balance. The sperm membrane is rich in polyunsaturated fatty acids (PUFAs), which are essential for preserving membrane fluidity, sperm motility, acrosome reactions, and sperm–egg fusion. Unfortunately, PUFAs are extremely susceptible to oxidation by ROS. Despite the presence of an antioxidant system in testis cells, an excess production of ROS beyond the reducing ability of the antioxidant system will trigger the peroxidation of PUFAs in the cell membrane, resulting in large amounts of lipid peroxides. These peroxides cause irreversible damage to sperm integrity, motility, and metabolic ability; damage sperm DNA integrity; and reduce semen quality [1]. Additionally, PUFAs can produce oxylipins via cyclooxygenase and lipoxygenase, which control testicular function by regulating immune function and inflammatory responses through autocrine and paracrine mechanisms.

Numerous studies have investigated the influence of season on semen quality in adult animals. Heat stress during summer exerts adverse effects on testicular development by reducing the number of spermatogenic cell layers in the seminiferous tubules, inducing vacuolar degeneration and atrophy of the seminiferous epithelium [2], and decreasing sperm quantity [3,4]. Compared with other seasons, bulls have a reduced relative number of spermatogenic cells in the seminiferous tubules in summer, accompanied with a decline in semen quality [5]. Additionally, seasonal effects on sperm in live-born animals may be associated with oxidative stress and autophagy [6]. Beyond influencing spermatogenesis and semen quality in the testes of sexually mature males, seasonal variations may also affect early testicular development. Studies have shown that animals born in summer have small testes and epididymides [7], whereas those born in autumn and winter exhibit early testicular development [8].

Hu sheep are the most widely distributed breed in China, distinguished by their early sexual maturity, high fertility, and estrus onset in all seasons. Spermatogenesis can occur in the testicular tissue of male lambs 84 days after birth, achieving sexual maturity at 6 months of age. However, little is known about the effects of seasonal factors on testicular development before sexual maturity in Hu rams. Therefore, in this study, testes were obtained from 6-month-old Hu sheep, born either in winter or summer, exhibiting similar growth and development under identical feeding conditions. By analyzing differences in cauda sperm density, antioxidant properties, oxylipins, and differentially expressed genes (DEGs) in testicular tissues between winter-born and summer-born rams, this study aimed to investigate whether different seasons affect sheep testicular development and the underlying mechanism.

## 2. Materials and Methods

### 2.1. Ethics Approval and Consent to Participate

All animal experiments were conducted in accordance with the ethical guidelines of the Animal Care and Use Committee of Lanzhou University (Ethic approval No. 2022013).

### 2.2. Sample Collection

The experiment was performed at Minqin Defu Agricultural Co., Ltd. All Hu sheep used in the experiment were fed the same commercial feed diet (Table S1) after being weaned at 56 days of age and raised until they reached 6 months of age. The winter-born group consisted of 18 6-month-old male Hu sheep lambs born in February, and the summer-born group included 18 lambs born in June. A total of 36 Hu lambs with similar body weight, testicular size, and in good health without any diseases were selected and slaughtered when reached sexual maturity stage at 6 months old in August 2022 and December 2022, respectively. The left testis tissue without tunica albuginea was collected; one part was fixed with animal testicular tissue fixation solution (Servicebio, Wuhan, China) for HE staining, and the other was preserved in liquid nitrogen.

Epididymal cauda sperm was collected using the swimming up method in accordance with our procedures described [9]. Briefly, the main part of the epididymal cauda was weighed and cut into small pieces and suspended with PBS. After incubation for 30 min at 37 °C, filtered out tissue pieces with a 200-mesh copper grid. Sperm was obtained and counted using a hemocytometer. The number of sperm was normalized with the weight of epididymal cauda tissue.

### 2.3. Testicular Histology

The fixed testis was dehydrated by increasing the alcohol concentration. The tissues were clarified with xylene, embedded in paraffin, and cut into 4 μm-thick sections for HE staining. Four images of different areas of each testis were captured by an Olympus IX71 microscope coupled with DP74 digital camera (Olympus, Tokyo, Japan), and 6–10 seminiferous tubules in each picture under a magnification of 100× were randomly chosen for diameter measurements. The thickness of the epithelium and the vacuolation in the spermatogenic epithelium were observed under a magnification of 200×.

### 2.4. Measurement of Antioxidant Properties

After grinding the testis tissues with liquid nitrogen, approximately 100 mg of testis tissue powder was mixed with 900 μL of PBS to prepare tissue homogenates. The suspension was collected by centrifugation at 3000 rpm for 10 min at 4 °C. The protein concentration was determined using a Bradford protein concentration assay kit (Biosharp Biotechnology, Hefei, China). The activity of superoxide dismutase (SOD), the content of malondialdehyde (MDA), and total antioxidant capacity (T-AOC) were measured at 450, 532, and 405 nm, respectively, using the kit (Nanjing Jiancheng Bioengineering Institute, Nanjing, China) in accordance with the manufacturer’s protocols. The concentrations of these antioxidant properties were normalized with the total protein concentration in testis tissue.

### 2.5. Quantitative Real-Time PCR Analysis of Antioxidant Genes

Total RNA was extracted using an RNA simple Total RNA Kit (TIANGEN Biotech, Beijing, China) following the manufacturer’s instructions. The integrity and purity of total RNA were assessed using agarose gel electrophoresis and NanoDrop 2000 (Thermo Fisher Scientific, Wilmington, NC, USA) spectrophotometer, respectively. Total RNA (0.5 μg) with OD260/OD280 between 1.9 and 2.1 was reverse-transcribed into cDNA using EasyScript^®^ One-Step gDNA Removal and cDNA Synthesis SuperMix (TransGen Biotech, Beijing, China) under 42 °C for 15 min. The cDNA was diluted 1:20 with nuclease-free water and stored at −80 °C. The relative mRNA expression levels of *GPX3*, *GPX4*, and *Cu*/*ZnSOD* were detected using the CFX384 Touch™ Real-Time PCR Detection System (Bio-Rad, Hercules, CA, USA) with the PerfectStart^®^ Green qPCR SuperMix (TransGen Biotech, Beijing, China). The reaction system comprised 10 μL of 2× perfectstart green qPCR SuperMix, 5 μL of cDNA, 0.4 μL of forward and reverse primers (Table 1), and 4.2 μL of DNase free water. The reaction procedure consisted of initial denaturation at 95 °C for 5 min, followed by 40 cycles of amplification (95 °C for 5 s and 61 °C for 30 s). The specificity of the primers was verified via melting curve analysis, and the relative quantification of each gene was normalized to *HPRT1* by using the 2^−ΔΔCT^ method.

### 2.6. Metabolomics Analysis of Oxylipins

Five individuals from each group were randomly selected for metabolomics analysis of oxylipins by Metware Biotechnology Inc. (Wuhan, China). Oxylipins were extracted from 20 mg of tissues with 80 μL of pure water and 200 μL of internal standard mixture of methanol/acetonitrile (1:1, *v*/*v*), as previously described [10]. After drying under a nitrogen stream, the residue was reconstituted in 120 μL of water/methanol (1:1, *v*/*v*) for analysis. Mix all samples equally in volume to prepare 3 quality control (QC) samples. During analysis, insert QC sample before 10 samples to examine the repeatability of the entire analysis process. An Ultra HPLC ExionLC™ AD coupled to a tandem mass spectrometer QTRAP^®^ 6500+ instrument (SCIEX, Framingham, MA, USA) with a Waters ACQUITY UPLC HSS T3 C18 column (1.8 µm, 100 mm × 2.1 mm i.d.) (WATERS, Framingham, MA, USA) was used for LC–MS/MS analysis. Mobile phase A was acetonitrile/water (60/40, *v*/*v*), and mobile phase B was acetonitrile/isopropanol (50/50, *v*/*v*). The flow rate was 0.4 mL/min, the column temperature was 40 °C, and the injection volume was 10 μL. The gradient profile was set as follows: 99.9% A from 0 min to 2 min, 70% A from 2 min to 4 min, 50% A from 4 min to 5.5 min, 1% A from 5.5 min to 7 min, and 99.9% A from 7 min to 7.1 min. Mass analysis was performed in electrospray ionization mode with an electrospray ion source temperature of 550 °C, ion spray voltage of −4500 V, and curtain gas pressure of 35 psi. In QTRAP 6500+, each ion pair was scanned and detected according to the optimized declustering voltage and collision energy. Quantitative analysis was performed using the multiple reaction monitoring mode of triple quadrupole mass spectrometry. Data acquisitions were performed using Analyst 1.6.3 software (Sciex). Multiquant 3.0.3 software (Sciex) was used to quantify all metabolites. Linear calibrations were built for each analyte by plotting the peak area ratio of analytes to internal standard (IS) versus molar amount ratio of analyte to IS (Table S2). The principal components analysis (PCA) and orthogonal least partial squares discriminant analysis (OPLS-DA) were performed by MetWare (http://www.metware.cn/, accessed on 23 January 2024) examine the relationship between metabolite expression profiles and sample groups. Differential oxylipins were identified with *VIP* ≥ 1 and |log_2_ fold change| > 1.

### 2.7. RNA Sequence

Total RNA was extracted from testes tissue, which were used for oxylipins analysis. The RNA integrity number and 28S/18S ratios were detected using an Agilent 2100 Bioanalyzer (Agilent Technologies, Santa Clara, CA, USA). Transcriptome library preparation and sequencing were performed on an Illumina NovaSeq platform, with 150 bp paired-end reads generated by Metware Biotechnology Inc. (Wuhan, China). Raw sequencing data were filtered using fastp to obtain clean reads, and the Q20, Q30, and GC contents were calculated. The clean reads were mapped to the ovine reference genome (NCBI RefSeq ID: GCF_016772045.1), and the mapped reads were quantified via FeatureCounts program (v2.0.3), which assigned reads to genomic features such as genes and exons specified in the reference file. A counts file was generated after filtering out genes with low expression (<10 counts across all groups). This file was used to identify DEGs via the DESeq2 R package (v1.22.1). Genes with |log_2_ fold change| ≥ 1 and *p* < 0.05 were assigned as DEGs. The expression level of each gene was estimated from fragments per kilobase of transcript per million mapped reads (FPKM). Various plots such as Heatmap, and Volcano plot were generated to depict the gene expression results. The hypergeometric test was used to analyze the enrichment of GO terms and KEGG pathways using the CLusterProfiler R package (v4.6.0). The GO terms and KEGG pathways with corrected *p* < 0.05 were considered to indicate the significant functional enrichment of DEGs.

### 2.8. Validation of Key Genes

The six genes (Table 1), including three upregulated and three downregulated genes, were validated by qRT-PCR.

### 2.9. Integrative Analysis

The quantitative values of genes detected by transcriptomics and oxylipins detected by LC–MS/MS in all samples were used for overall correlation analysis. Correlations with an absolute correlation coefficient > 0.8 and *p* < 0.05 were selected, and the fold differences between genes and oxylipins corresponding to these correlations were illustrated using a nine-quadrant diagram. The correlation results were imported into Cytoscape 3.9.0 to construct the network.

### 2.10. Statistical Analyses

The parameters of body weight, scrotum circumference, testis weight and index, testicular histology index, antioxidant properties, sperm density, and qRT-PCR were expressed as “mean ± standard errors” and evaluated with the SPSS program 13.0 (SPSS, Chicago, IL, USA). Normal parameters were analyzed by the one-sample Kolmogorov–Smirnov test. The homogeneity of variances was analyzed by Levene’s test. Significant differences between the two groups were analyzed by Student’s *t*-test and Welch’s *t*-test for unequal variances. The results were visualized with GraphPad Prism 8.2.1.441 (GraphPad Software, San Diego, CA, USA). Effect sizes were calculated using Partial Eta-square (η^2^). Further comparative statistical analysis of oxylipins and transcriptome were conducted on Metware cloud (https://cloud.metware.cn/, accessed on 23 January 2024).

## 3. Results

### 3.1. Differences in Reproductive Organ Parameters Between the Winter-Born and Summer-Born Groups

No significant differences were observed in the body weight, testis weight, scrotum circumference, and testis index between the two groups (*p* > 0.05, Table 2). However, the cauda sperm density in the winter-born group was significantly lower than that in the summer-born group (*p* < 0.001).

### 3.2. Histological Observations

**Figure 1:**
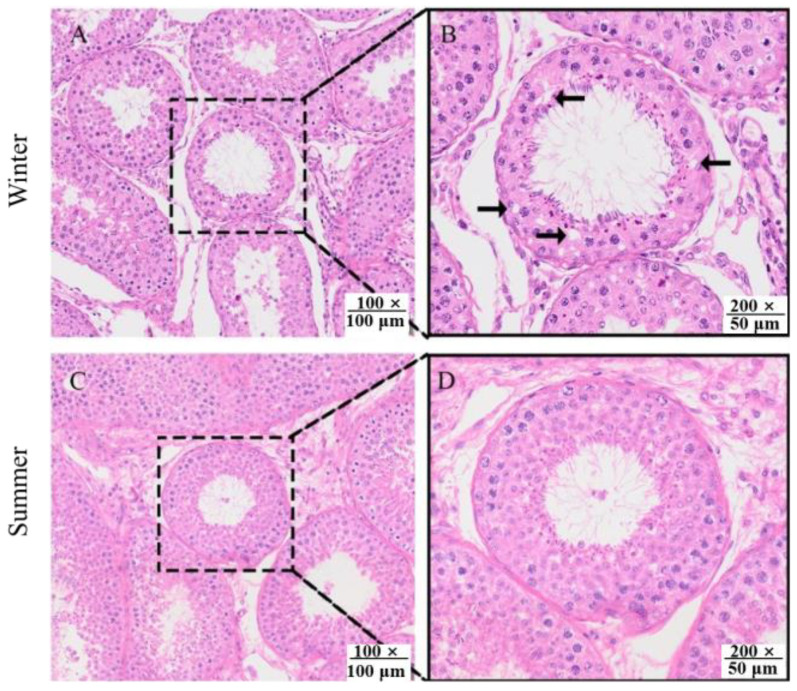
Histological images of testis from the winter-born group (**A**,**B**) and summer-born (**C**,**D**) group. Multiple change was presented in the black box (200×). The vacuolation in the spermatogenic epithelium was indicated by the black arrow.

The thickness of the epithelium in the summer-born group was significantly higher than those in the winter-born group (*p* = 0.01, Table 2). The diameter of seminiferous tubules were showed an upward trend in the summer-born group (*p* = 0.07). Additionally, vacuolation was observed in the spermatogenic epithelium of the winter-born group (Figure 1).

### 3.3. Effect of Season on Antioxidant Properties

**Figure 2:**
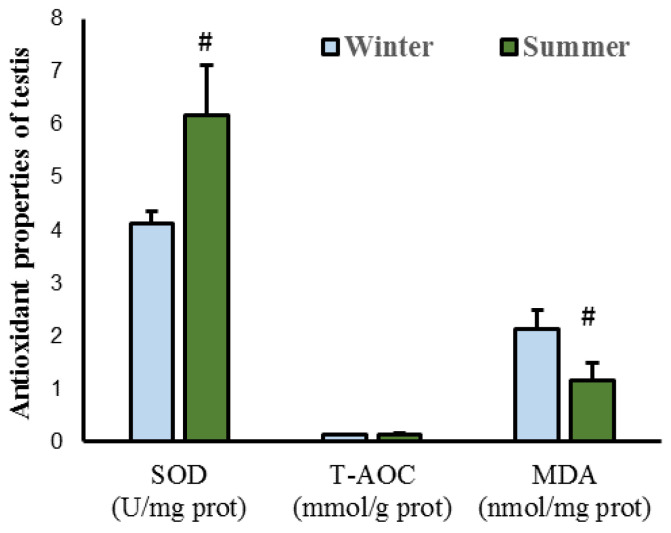
Antioxidant properties of testis from the winter-born group and summer-born group. Compared with the winter-born group, the activity of superoxide dismutase (SOD) was significantly increased, whereas the malondialdehyde (MDA) content was decreased in the summer-born group. There was no difference in total antioxidant capacity (T-AOC) levels between the two groups. # represents *p* < 0.05.

**Figure 3:**
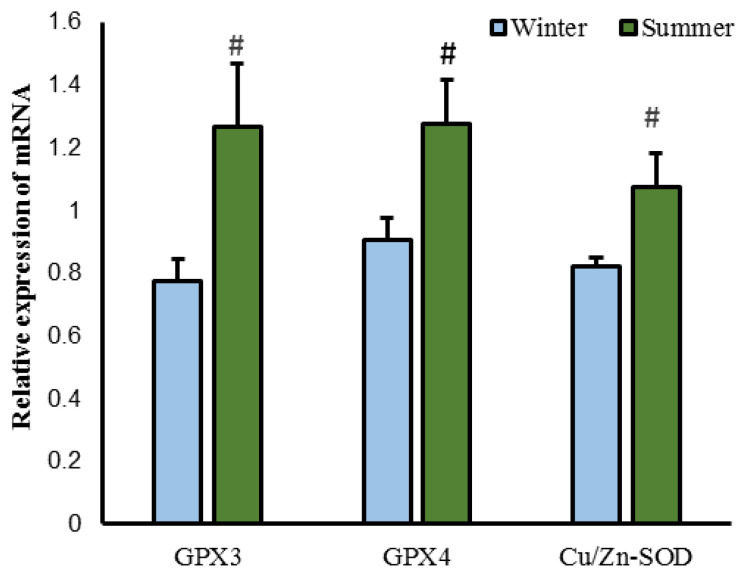
Relative expression levels of antioxidant-related genes between the winter-born group and summer-born group. The glutathione peroxidase 3 (*GPX3*), glutathione peroxidase 4 (*GPX4*), and copper/zinc superoxide dismutase (*Cu*/*Zn-SOD*) genes were significantly higher in the summer-born group than in the winter-born group. # represents *p* < 0.05.

As shown in Figure 2, the testicular SOD activity in the winter-born group was significantly lower than that in the summer-born group (*p* < 0.05), whereas the MDA content was significantly higher (*p* < 0.05). We found no significant difference in the level of T-AOC between the two groups (*p* > 0.05). The relative expression levels of *GPX3*, *GPX4*, and *CU*/*Zn-SOD* in the testes of the winter-born group were significantly lower than those in the summer-born group (*p* < 0.05, Figure 3).

### 3.4. Metabolic Analysis of Oxylipins

**Figure 4:**
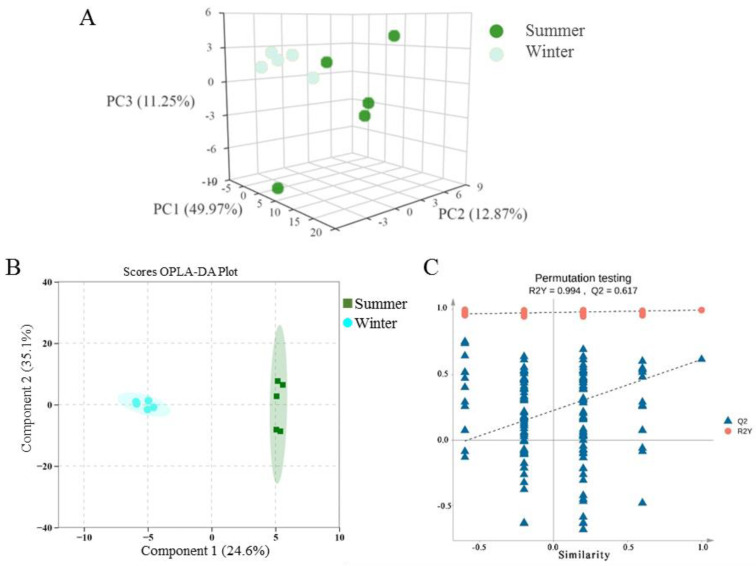
The principal components analysis and orthogonal least partial squares discriminant analysis between two groups. (**A**) 3D diagram of grouping principal component analysis. (**B**) OPLS-DA score plot and permutation testing of the OPLS-DA model (**C**) between two groups.

Three QC samples were used to analyze the stability of the quality of metabolomics data. Pearson correlation coefficient among QC samples ranged from 0.9989 to 0.9993. PCA (Figure 4A) and OPLS-DA results revealed significant differences in oxylipin profiles between the testes of winter-born and summer-born sheep. PC1, PC2 and PC3 accounting for 49.97%, 12.87% and 11.25% of the variance, respectively. The model showed good predictive capability with R^2^Y = 0.994, Q2 = 0.617, respectively (Figure 4B,C).

**Figure 5:**
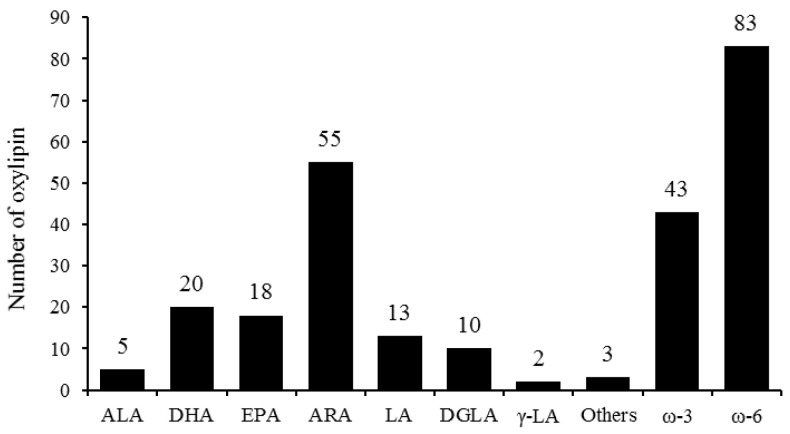
Number of oxylipins detected in the testis. A total of 126 oxylipins were detected, which were derived from α-linolenic acid (ALA), docosahexaenoic acid (DHA), eicosapentaenoic acid (EPA), arachidonic acid (ARA), linoleic acid (LA), dohomo-γ-linolenic acid (DGLA) γ-LA and other. Among them, the total number of oxylipins derived from ω-6 polyunsaturated fatty acid (PUFA) was higher than those derived from ω-3 PUFA.

**Figure 6:**
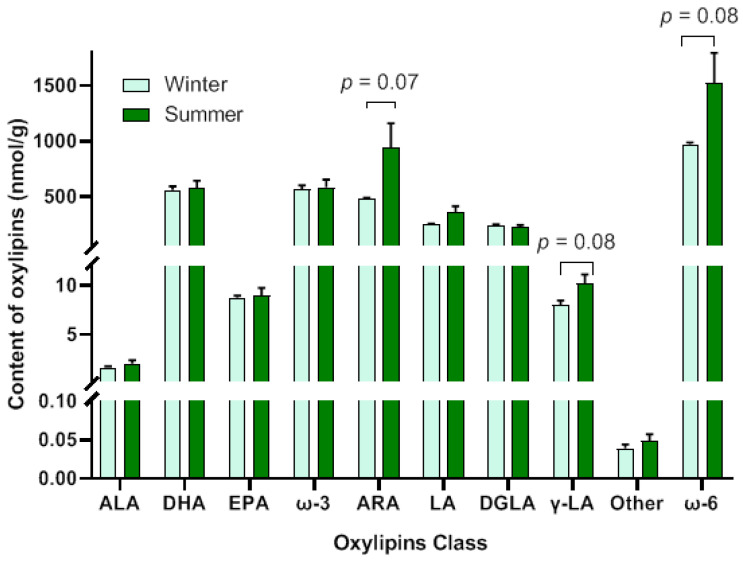
Content of oxylipin detected in the testis between two groups. Compared to the winter-born group, the content of oxylipins derived from arachidonic acid (ARA), γ-linoleic acid (γ-LA) and ω-6 polyunsaturated fatty acid (PUFA) showed an upward trend in the summer-born group. However, there were no differences in α-linolenic acid (ALA)-, docosahexaenoic acid (DHA)-, eicosapentaenoic acid (EPA)-, LA-, dohomo-γ-linolenic acid (DGLA)- and ω-3 PUFA-derived oxylipins between two groups.

A total of 126 oxylipins were detected in this experiment (Table S3), which were classified into 8 major categories: α-linolenic acid (ALA), docosahexaenoic acid (DHA), eicosapentaenoic acid (EPA), arachidonic acid (ARA), linoleic acid (LA), dohomo-γ-linolenic acid (DGLA), γ-LA, and others (Figure 5). The number of oxylipins derived from ω-6 PUFA accounted for 65.87% of the total number of oxylipins, among which ARA-derived oxylipins were the most abundant, representing 43.65% of the total. The number of oxylipins derived from DHA was the largest in ω-3 PUFA-derived oxylipins, accounting for 15.87% of the total number of oxylipins. Compared with the winter-born group, the detection results of the oxylipin content showed that only ARA-(*p* = 0.07), γ-LA-(*p* = 0.08) and ω-6 PUFA-derived (*p* = 0.08) oxylipins showed an upward trend in the summer-born group (Figure 6).

### 3.5. Identification of Differential Oxylipins

**Figure 7:**
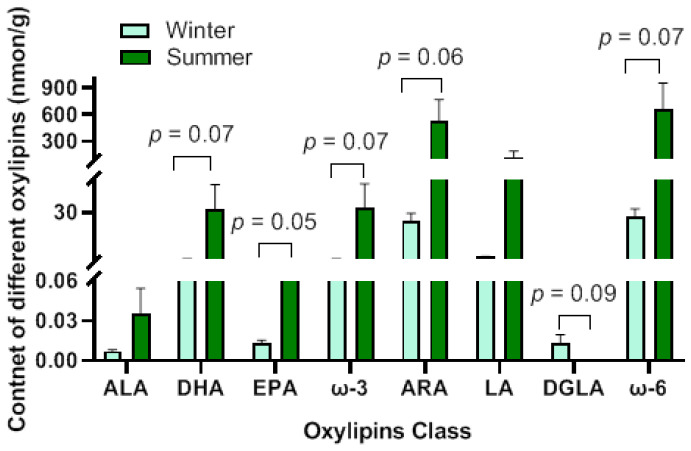
Content of differential oxylipins in the testis between two groups. In summer-born group, the total content of differential oxylipins derived from eicosapentaenoic acid (EPA) was significantly evaluated. The total content of differential oxylipins derived from arachidonic acid (ARA), docosahexaenoic acid (DHA), ω-3 polyunsaturated fatty acid (PUFA) and ω-6 PUFA showed upward trend than those of the winter-born group. However, the total content of differential oxylipins derived dohomo-γ-linolenic acid (DGLA) was showed a downward trend in summer-born group. There was no difference in α-linolenic acid (ALA)- and linoleic acid (LA)-derived oxylipins between two groups.

A total of 44 differential oxylipins were identified, including 36 upregulated and 8 downregulated in the summer-born group (Table 3). Among them, 26 oxylipins were derived from ω-6 PUFA, accounting for 59.09% of the total differential oxylipins, with ARA-derived ones being the most numerous (43.18% of the total). The EPA-derived oxylipins comprised the largest proportion of all oxylipins derived from ω-3 PUFA, accounting for 20.45% of the total. No significant differences were observed in the total content of differential oxylipins derived from ALA (*p* = 0.16) and LA (*p* = 0.1). The total content of differential oxylipins derived from EPA was significantly elevated in the summer-born group (*p* = 0.05). The total content of differential oxylipins derived from ARA (*p* = 0.06), DHA (*p* = 0.07), ω-3 PUFA (*p* = 0.07) and ω-6 PUFA (*p* = 0.07) were showed an upward trend in the summer-born group. However, the total content of differential DGLA-derived oxylipins showed a downward trend in the summer-born group (*p* = 0.09) (Figure 7).

### 3.6. Analysis of RNA Sequencing Data

**Figure 8:**
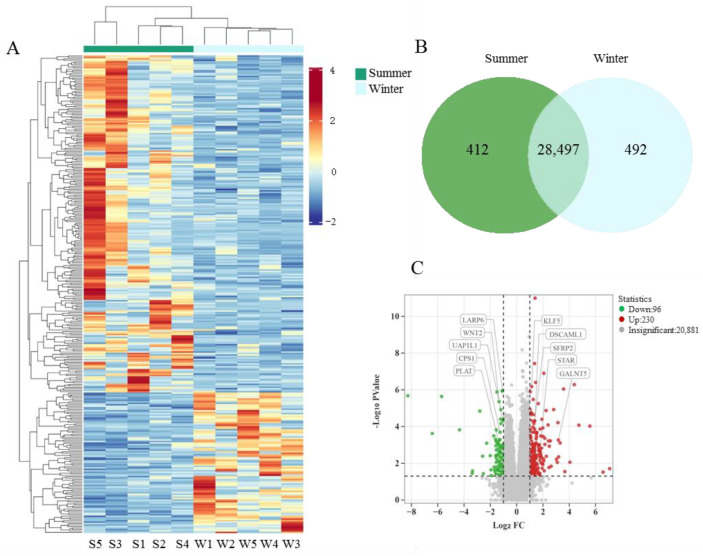
Analysis of RNA sequencing data. Hierarchical clustering analysis revealed a clear distinction between the two groups (**A**). There were 28,497 genes co-expressed in both groups (**B**). There were 326 differentially expressed genes screened between two groups (**C**).

Ten libraries (five per group) were sequenced through Illumina HiSeq sequencing, and the raw read counts ranged from 53,319,680 to 91,731,658. Over 51,848,386 clean reads were generated after the adapters in reads were removed and low-quality sequences were filtered. The percentage of clean reads above 20% (Q20) from all samples surpassed 97.76%, whereas Q30 was above 93.79%. These results indicated that the quality of the database was satisfactory. About more than 92.19% of clean reads were uniquely mapped to the reference genome, which was above the minimum requirement of 70% (Table S4). Hierarchical clustering analysis revealed a clear distinction between the two groups, with relatively small within-group variation (Figure 8A). A total of 412 genes were specifically expressed in the summer-born group, 492 were uniquely expressed in the winter-born group, and 28,497 genes were co-expressed in both groups (Figure 8B). Among them, 230 DEGs were upregulated and 96 DEGs were downregulated in the summer-born group with |log_2_ fold change| ≥ 1 and *p* < 0.05 (Table S5, Figure 8C).

### 3.7. GO and KEGG Analyses of DEGs

**Figure 9:**
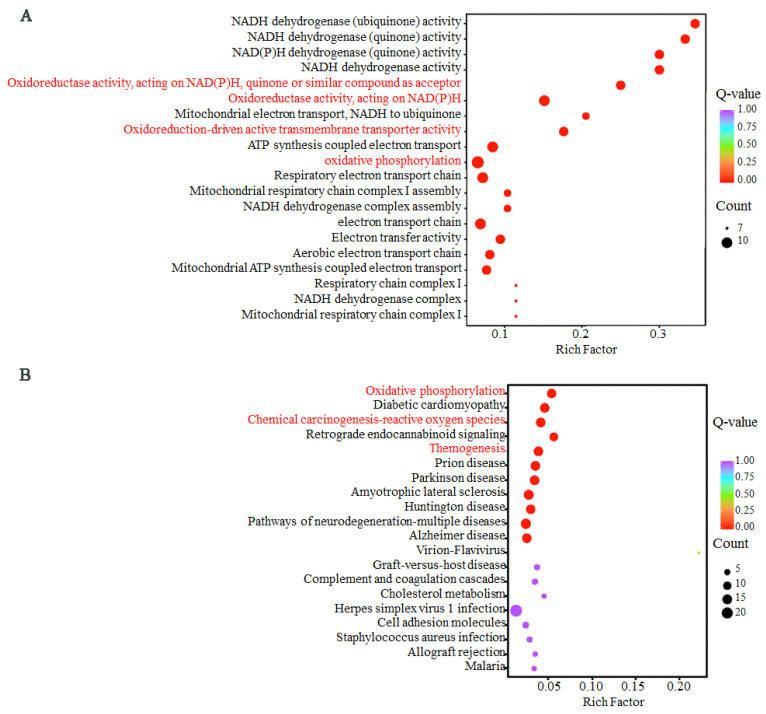
Gene ontology (**A**) and Kyoto encyclopedia of genes and genomes (**B**) enrichment of differentially expressed genes of testis between the winter-born and summer-born groups.

The DEGs were assigned to 35 GO terms, including two biological processes, 12 cellular components, and 21 molecular functions, with ≥3 associated gene numbers identified. Terms related to antioxidant activity, such as oxidoreductase activity acting on NAD(P)H, quinone or similar compounds as acceptors, oxidoreduction-driven active transmembrane transporter activity, oxidoreductase activity acting on NAD(P)H), and oxidative phosphorylation, were enriched (Figure 9A). KEGG enrichment analysis revealed the DEGs related to metabolic and antioxidant functions, include the metabolic pathway, oxidative phosphorylation, chemical carcinogenesis–ROS, and thermogenesis (Figure 9B).

### 3.8. Verification of DEGs by qRT-PCR

**Figure 10:**
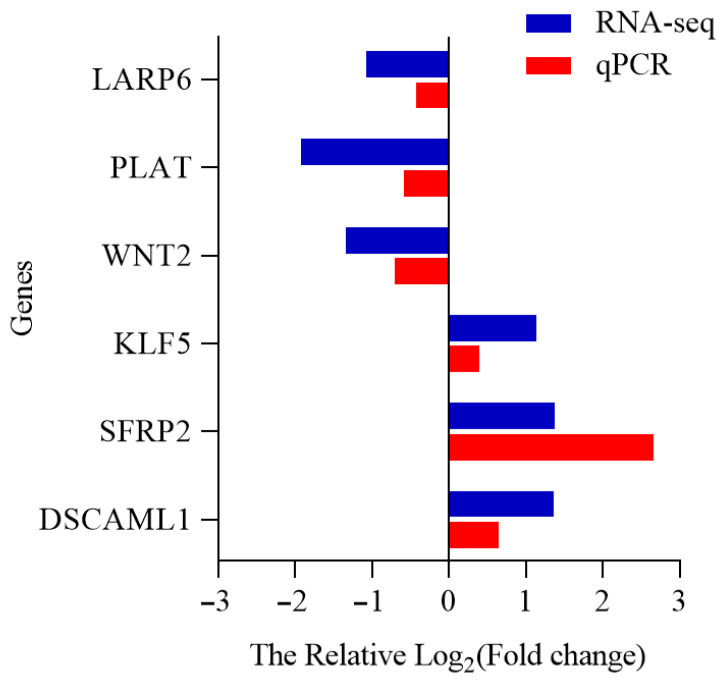
Validation of the expression levels of differentially expressed genes by qRT-PCR. The expression level of the six genes showed a pattern that correlated well with that of the sequencing data. The fold change was calculated as the ratio of summer values to winter values (summer/winter).

The expression levels of six DEGs (*DSCAML1*, *SFRP2*, *WNT2*, *KLF5*, *LARP6* and *PLAT*) were analyzed using qRT-PCR, and the results showed a good correlation with the sequencing data (Figure 10).

### 3.9. Combined Analysis of Transcriptomics and Oxylipidomics

**Figure 11:**
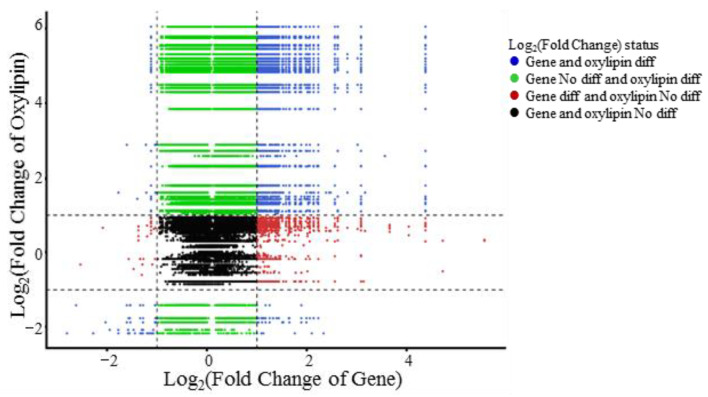
Nine-quadrant diagram of oxylipin and gene correlation analysis. Note: Each dot represents a pairwise correlation between oxylipins and genes, from left to right, top to bottom, in quadrants 1–9. Quadrants 1 and 9 represent genes with opposite patterns of differential expression to oxylipins; quadrants 3 and 7 represent the same pattern of expression; quadrant 5 represents that neither the gene nor the oxylipin is differentially expressed; and quadrants 2, 4, 6, and 8 represent that the expression of oxylipins is unchanged, genes are up- or downregulated, or that the gene is not changed in expression that oxylipins are up- and downregulated.

Overall correlation analysis was performed on the quantitative values of genes identified by transcriptomics and oxylipins detected by LC–MS/MS in all samples. A nine-quadrant diagram (Figure 11) displayed the fold differences of the genes and oxylipins corresponding to the correlation relationships with an absolute correlation coefficient > 0.8 and *p* < 0.05.

**Figure 12:**
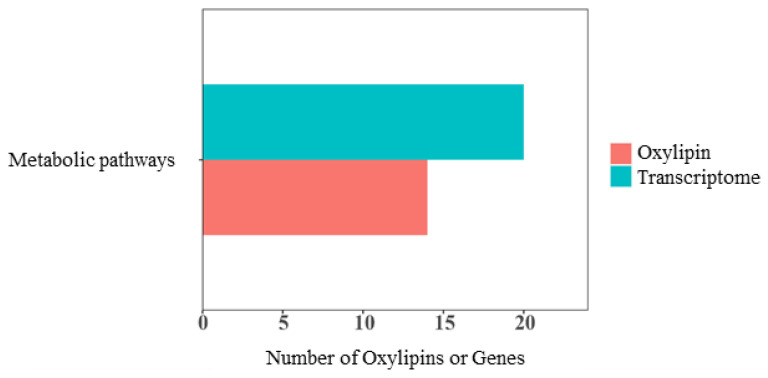
Bar graph of KEGG enrichment analysis.

**Figure 13:**
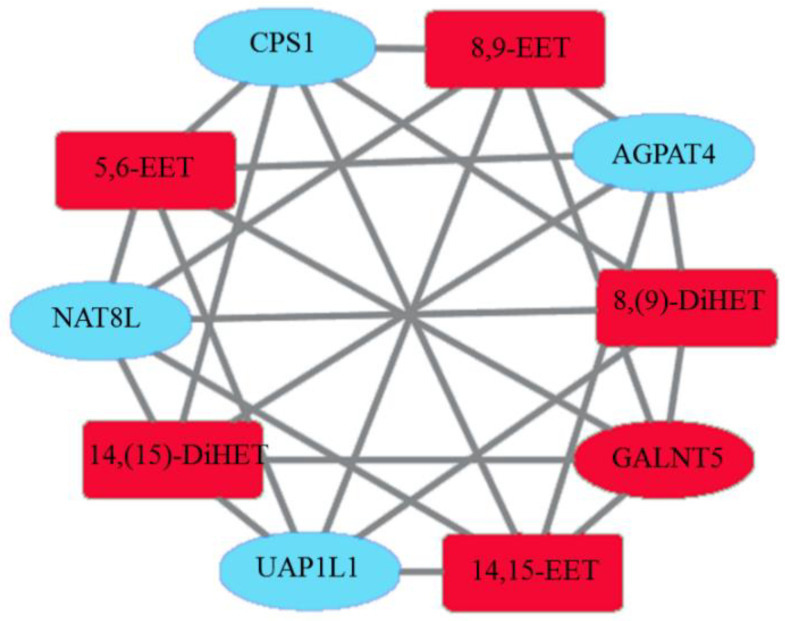
Correlation network diagram of differentially expressed genes and differential oxylipins. Note: Blue represents downregulation, red represents upregulation, ovals represent differentially expressed genes, and rectangles represent differential oxylipins.

KEGG analysis showed that differential oxylipins and DEGs were enriched in metabolic pathways (Figure 12). Fourteen different oxylipins such as 8(9)-DiHET, 8,9-EET, and PGD2 were enriched in the metabolic pathway, and 14 DEGs such as *UAP1L1*, *CPS1*, and *GALNT5* were enriched. The correlation network diagram revealed a significant correlation between *NAT8L* genes and 14,15-EET, 14(15)-DiHET, and 8(9)-DiHET oxylipins. A significant negative correlation was observed between *UAP1L1* and 8,9-EET, 5,6-EET, 14,15-EET, 14(15)-DiHET, and 8(9)-DiHET. A consistent regulatory relationship existed between genes (*NAT8L* and *UAP1L1*) and different oxylipins (8,9-EET, 5,6-EET, 14,15-EET, 14(15)-DiHET, and 8(9)-DiHET). These results indicated that the expression of DEGs was correlated with the biosynthesis of differential oxylipins, and their interaction may affect testicular development in Hu sheep (Figure 13).

## 4. Discussion

This study aimed to investigate the influence of birth season on sheep testis development before sexual maturity. To minimize the effect of factors such as body weight, and testis size on the results, we selected 6-month-old Hu sheep with similar body weight, scrotal circumference, and testis weight for the research. However, in the summer-born group, the sperm density in epididymis cauda was significantly higher than that in the winter-born group. This result was consistent with previous research [2]. In contrast to winter, high temperatures in summer might disrupt the antioxidant balance, leading to the accumulation of ROS and adversely affecting testicular development. This may lead to a reduction in the layers of spermatogenic cells within the seminiferous tubules and induce vacuolar degeneration and atrophy of the spermatogenic epithelium. In this study, the thickness of the epithelium in the winter-born testis significantly decreased, and some vacuolation was also observed in the spermatogenic epithelium. SOD is critical to the antioxidant response in mammals and serves as the primary defense mechanism within the antioxidant enzyme defense system [11], whereas MDA is a byproduct of lipid peroxidation reactions. In this study, SOD activity significantly increased and the MDA content decreased in the summer-born group. The mRNA expression levels of antioxidant-related genes *GPX3*, *GPX4*, and *Cu*/*Zn-SOD* also increased significantly. Thus, the diminished antioxidant capacity and elevated degree of lipid peroxidation in the winter-born testicular tissue were responsible for the reduction in spermatogenesis [12].

Oxylipins are a series of oxidized metabolites derived from PUFAs through auto-oxidation or through the enzymatic activity of cyclooxygenase (COX), lipoxygenase (LOX), and cytochrome P450 (CYP450). The COX pathway forms prostaglandins (PGs) and thromboxanes (TXs); the LOX pathway produces leukotrienes and lipoproteins; and the CYP450 pathway generates various epoxy, hydroxyl, and dihydroxy derivatives [13]. As small metabolites downstream of the biological system, oxylipins metabolites play significant roles in the body’s homeostasis and disease states. They are involved in processes such as redox balance, oxidative stress, signal transduction, iron poisoning, and inflammation, thereby participating in the regulation of testicular function.

In this study, differential oxylipins were screened by using LC-MS/MS, and 44 differential oxylipins were identified, nearly half of which were derived from ARA. CYP450 catalyzes the metabolism of free ARA into epoxyeicosatrienoic acid (EET) [14]. The results of KEGG enrichment analysis showed that the ARA metabolism pathway exhibited the highest number of differentially enriched oxylipins, including 5,6-EET, 8,9-EET, 11,12-EET, 14,15-EET, 8(9)-DiHET, 11(12)-DiHET, 14(15)-DiHET, 5,6-DiHETrE, prostaglandin D2 (PGD2), and lipoxin B4 (LXB4). EETs may be involved in maintaining cellular antioxidant capacity. In human, the decrease in 14,15-EET concentration leads to reduced antioxidant capacity in granulosa cells, decreased ATP production, thus, stimulating the ROS accumulation in oocytes and resulting in diminished fertility. Fortunately, 14, 15-EET treatment could alleviate granulosa cell senescence and improve fertility by inhibiting excessive activation of the PI3K/AKT/mTOR signaling pathway [15]. In this study, the concentration of 5,6-EET, 8,9-EET, 11,12-EET, 14,15-EET were all significantly upregulated with the stronger anti-oxidant capacity in the summer-born testis. However, EETs are highly unstable and rapidly metabolized. Their main metabolic pathway is the conversion into dihydroxyeicosatrienoic acids (DHETs) under the catalysis of soluble epoxide hydrolase [16]. In addition to their anti-oxidant ability, previous studies also indicated that olylipins such as EETs, DHETs, DiHETrE and PGD2 can activate the peroxisome proliferator-activated receptor alpha (PPARα) pathway and exhibit anti-inflammatory activity and vasodilatory effects [17]. The concentration of PGD2 was decreased in the hippocampus and cortex of offspring rats exposed to maternal lipopolysaccharide, in parallel with increased oxidative stress and inflammation [18]. In this study, the thickness of the epithelium and the cauda sperm density and in summer-born group were significantly higher than in the winter-born group. Furthermore, the inflammatory lipid mediators PGF1α [19] and oxidative stress mediator 5-isoPGF2VI [20] were significantly downregulated in the testis of summer-born group. As an anti-inflammatory lipid mediator, the content of LXB4 [21] was significantly increased in the summer-born group. These findings indicated that the testicular tissue of the summer-born rams exhibited enhanced anti-inflammatory activity and reduced oxidative stress with the content of MDA significantly decreased, which contributed to spermatogenesis.

Transcriptomic analysis identified 326 DEGs between the two groups. GO and KEGG enrichment analyses revealed that several DEGs including *WNT2*, *SFRP2*, *CPS1*, *UAP1L1*, and *GALNT5*, were enriched in pathways related to metabolism and oxidation functions. Thus, these DEGs may significantly influence the seasonal impacts on testicular tissue. *WNT2* can enhance the activation of the JAK-STAT pathway in male germ cells. The continuous activation of the JAK-STAT signaling pathway is closely related to many biological processes such as apoptosis, cell proliferation and differentiation, and oxidative stress, especially spermatogenesis [22]. In testes with ischemia–reperfusion injury, JAK2/STAT3 activity was increased with anti-oxidant ability decreased, and spermatogenic arrest [23]. In this study, the downregulation of *WNT2* in the summer-born testis was beneficial for preventing oxidative stress via inhibiting the JAK-STAT signaling pathway and provided a favorable microenvironment for normal spermatogenesis. *SFRP2* exhibits anti-apoptotic [24] and anti-oxidative stress [25] properties. PGC1-α is a key regulatory factor of oxidative metabolism, and it helps maintain mitochondrial biogenesis and function [26]. Studies have found that the overexpression of *SFRP2* leads to elevated expression levels of PGC1-α. *SFRP2* improves mitochondrial function through the AMPK–PGC1-α axis, thereby protecting cells from oxidative stress and apoptosis induced by glycolipid toxicity in vivo and in vitro [27]. The upregulation of *SFRP2* gene expression in the summer-born group enhances antioxidant capacity and reduces the risk of oxidative stress. *CPS1* is the rate-limiting enzyme that controls the first reaction of the urea cycle and belongs to the glutamine aminotransferase class [28]. The deficiency of *CPS1* promotes the synthesis of some lipid molecules [29], whereas the overexpression of *CPS1* is associated with poor prognosis in various cancers, such as colon cancer, cholangiocarcinoma, and glioblastoma [30]. The downregulation of *CPS1* gene expression in the summer-born rams facilitated the synthesis of lipid molecules and provided a relatively stable environment for spermatogenesis. *CDCA8* is a member of the cell cycle division-related protein family [31] and serves as a key regulator of mitosis and cell division. It is upregulated in many types of cancer tissues but diminished or absent in normal tissues [32]. The overexpression of *UAP1L1* enhances the proliferation and migration of prostate cancer cells but reduces the inhibitory effect of *CDCA8* knockdown on prostate cancer development [33]. The downregulation of *UAP1L1* in summer-born testicular tissue could prevent excessive cell proliferation and the accumulation of elevated levels of ROS, providing a safe environment for spermatogenesis. *GALNT5* is the enzyme that initiates mucin-type O-glycosylation. Studies have shown that *GALNT5* is significantly downregulated during ferroptosis [34]. Ferroptosis is a form of programmed cell death induced by iron accumulation and iron-dependent lipid peroxidation [35]. Ferroptosis-inducing factors can directly or indirectly affect glutathione peroxidase, leading to a decrease in antioxidant capacity and the accumulation of lipid ROS in cells, ultimately resulting in cellular oxidative death. *GPX4* plays a key role in ferroptosis, and it can convert the key intermediate lipid hydroperoxide into lipid alcohol to effectively prevent lipid peroxidation damage [36,37]. The upregulation of *GPX4* and *GALNT5* expression levels in the testicular tissue of summer-born rams indicated enhanced antioxidant capacity and reduced lipid peroxidation in their testicular tissue.

Combined correlation analysis of DEGs and differential oxylipins, along with KEGG enrichment analysis, revealed that both omics datasets were enriched in metabolic pathways. Within these metabolic pathways, significant correlations were observed between DEGs and oxylipins, specifically between *NAT8L* and 8,9-EET, 5,6-EET, 14,15-EET, 14(15)-DiHET, and 8(9)-DiHET; as well as between *UAP1L1* and 8,9-EET, 5,6-EET, 14,15-EET, 14(15)-DiHET, and 8(9)-DiHET. *NAT8L* and *UAP1L1* showed a consistent regulatory relationship with these differential oxylipins. They were negatively correlated with 8,9-EET, 5,6-EET, 14,15-EET, 14(15)-DiHET, and 8(9)-DiHET. NAT8L can catalyze acetyl-CoA and aspartic acid to form the N-acetyl-L-aspartic acid (NAA) pathway. The overexpression of *NAT8L* leads to excessive cell proliferation and increased oxygen consumption [38], which are not conducive to spermatogenesis. In this study, the downregulation of *NAT8L* and *UAP1L1* in the testis of summer-born specimens could prevent excessive cell proliferation and reduce ROS accumulation, providing a good microenvironment for spermatogenesis. These results indicated that the downregulation of DEGs related to unfavorable spermatogenesis (such as *UAP1L1* and *NAT8L*) in the testicular tissue of summer-born rams was significantly associated with the upregulation of EETs and DiHETs. These factors worked synergistically to reduce oxidative stress, thereby providing an enhanced microenvironment for spermatogenesis.

This study has some limitations that should be considered. First, the stability of the reference gene *HPRT1* of this experiment was not systematically validated. We selected *HPRT1* as a single reference gene primarily based on its well-documented stability in previous studies focusing on testicular development in Hu sheep [9]. It is widely recognized that reference gene stability can be influenced by experimental conditions. We note that our qPCR validation of 6 DEGs selected from the transcriptomic exhibited consistent expression trends with the transcriptomic results. This consistency provides indirect support for the reliability of *HPRT1* as a reference gene in the current experimental system. To address this limitation in future research, it would be ideal to screen and validate multiple candidate reference genes, or at least three reference genes need to be included according to MIQE Guidelines.

Another important limitation that we did not apply false discovery rate (FDR) to reduce the risk of false positives for multi-omics analysis. We acknowledge the importance of FDR correction in controlling the false discovery rate for large-sample studies. For oxylipins, there were only 141 oxylipins in the pools and 126 oxylipins were detected in our experiment, thus the relatively small number of tests reduced the potential for false positives. The number of individuals was really small, and the differences among individuals within a group might lead to a higher *p* value level. Therefore, the differential oxylipins were identified based on |log_2_ fold change| > 1 and the VIP value ≥ 1 as reported in previous studies [39]. For transcriptomic studies, the approach of screening DEGs using raw *p*-values combined with fold change was widely accepted [40]. This method also offered higher sensitivity in detecting potential inter-group expression differences. In the present study, we employed the conditions of *p* ≤ 0.05 and a relatively strict fold change threshold (|log_2_ fold change| ≥ 1) to identify DEGs, resulting in the identification of 316 DEGs. For correlation analysis, r > 0.8 not only cuts false positives but also represents likely direct biological functional links. In this study, r > 0.8 corresponds to *p* ≈ 0.005 (far below 0.05), so FDR correction was unnecessary. With more than one hundred tests, FDR’s value expansion may wrongly exclude key associations (e.g., ARA-*ALOX*: r = 0.8099, *p* = 0.0046, FDR = 0.1128; 9-HETE-*DAGLA*: r = −0.8223, *p* = 0.0035, FDR = 0.1007), conflicting with our goal of identifying reliable regulatory relationships. Our criteria balance rigor and biology, fitting oxylipin-gene association goals. In future studies, to enhance the reliability of the results, we should increase the sample size and adopt a FDR-controlling strategy to identify DEGs or metabolisms.

## 5. Conclusions

Compared with winter-born rams, summer-born rams exhibit stronger antioxidant capacity and lower lipid peroxidation in their testicular tissue at sexual maturity stage. Combined analysis of lipidomics and transcriptomics showed that the downregulation of DEGs such as *UAP1L1* and *NAT8L* was significantly associated with the upregulation of differential oxylipins such as EETs and DiHETs. These factors work synergistically to contribute to spermatogenesis.

## Figures and Tables

**Table 1:** Primer sequence information.

Genes	Primer Sequence (5′–3′)	Product Size (bp)
*GPX3* XM_015096153.3	F: TCCATGACATCCGGTGGAAC R: GCATGGGAGTGTGGCATAGT	250
*GPX4* XM_060416444.1	F: CCACCCTCTGTGGAAATGGAT R: GAGGGACGACTTTTCCCGA	195
*Cu*/*Zn-SOD* XM_004012626.3	F: GGAGACCTGGGCAATGTGAA R: CCTCCAGCGTTTCCAGTCTT	137
*HPRT1* XM_015105023.2	F: CGACTGGCTCGAGATGTGAT R: TCACCTGTTGACTGGTCGTT	197
*DSCAML1* XM_027979838.2	F: GCTCCCAGCATGGTGTTACT R: CGGTGATCTCAAACTTGGCG	189
*SFRP2* NM_001163053.1	F: ACAACGACCTTTGCATCCCC R: CCTTCTCGGACACTCCGTTC	249
*WNT2* NM_001195319.1	F: GGTCAGCTCTTCATGGTGGT R: CCAATGGCACGCATCACATC	132
*KLF5* XM_004012185.5	F: CAGTGCCTCAGTCGTAGACC R: GGCCAGTTCTCAGGTGAGTG	104
*LARP6* XM_027971542.3	F: GCTGGTGGACCAGATCGAAT R: AGGTGAGCAGCTTGACACTC	116
*PLAT* XM_012106011.3	F: GAATAGGGGTTATGGGGCGG R: CAGCATGTTGTTGGTGACGG	137

**Table 2:** Sample statistics.

Groups	Winter	Summer	*p* Value	95% Confidence Interval	η^2^
Body weight (kg)	47.23 ± 1.74	47.60 ± 1.18	0.86	−4.75–4.01	0.0009
Scrotum circumference (cm)	25.95 ± 0.35	26.81 ± 0.50	0.17	−2.10 to 0.39	0.05
Testis weight (g)	241.51 ± 2.68	239.16 ± 2.76	0.55	−5.53–10.22	0.01
Testis index (g/kg)	5.11 ± 0.17	5.02 ± 0.13	0.9995	−0.42–0.45	0.0001
Sperm density (10^7^/g)	16.86 ± 2.02	102.65 ± 9.56	<0.001	−105.4–−65.80	0.69
Seminiferous tubule diameter (μm)	208.69 ± 2.59	219.22 ± 4.84	0.07	−7.88–−1.17	0.31
Thickness of the epithelium (μm)	69.36 ± 1.12	73.88 ± 1.14	0.01	−22.07–1.02	0.17

Note: Effect sizes were calculated using Partial Eta-square (η^2^).

**Table 3:** Information of differential oxylipins.

No.	Differential Oxylipins	Classification	VIP	*p* Value	Fold Change	Type
1	9(S)-HpOTrE	ALA	1.01	0.20	4.92	up
2	8(9)-DiHET	ARA	1.16	0.13	29.84	up
3	8,9-EET	ARA	1.25	0.10	22.32	up
4	13,14-dihydro-15-ketoPGD2	ARA	1.41	0.07	0.00	down
5	PGD2	ARA	1.10	0.13	6.03	up
6	TXB2	ARA	1.04	0.13	3.46	up
7	5-isoPGF2VI	ARA	1.39	0.05	0.24	down
8	13,14-dihydro-15-ketoPGF2α	ARA	1.41	0.02	0.22	down
9	5,6-EET	ARA	1.26	0.09	21.06	up
10	8(S),15(S)-DiHETE	ARA	1.62	0.01	0.29	down
11	5(S),15(S)-DiHETE	ARA	1.50	0.04	0.37	down
12	14(15)-DiHET	ARA	1.17	0.13	53.55	up
13	14,15-EET	ARA	1.23	0.10	28.32	up
14	12-HHT	ARA	1.76	0.01	0.27	down
15	5,6-DiHETrE	ARA	1.34	0.06	22.42	up
16	11-keto-TXB2	ARA	1.10	0.11	2.82	up
17	LXB4	ARA	1.04	0.18	Inf	up
18	17-HETE	ARA	1.72	0.02	3.03	up
19	11,12-EET	ARA	1.25	0.09	19.57	up
20	11(12)-DiHET	ARA	1.16	0.13	32.73	up
21	PGF1α	DGLA	1.45	0.08	0.00	down
22	PGD1	DGLA	1.17	0.19	0.00	down
23	7(8)-DiHDPE(A)	DHA	1.19	0.12	Inf	up
24	19(20)-EpDPE(A)	DHA	1.20	0.12	43.59	up
25	19(20)-DiHDPE(A)	DHA	1.16	0.13	31.00	up
26	7,8-EpDPE	DHA	1.25	0.10	29.27	up
27	16(17)-EpDPE	DHA	1.24	0.10	36.35	up
28	PDX	DHA	1.00	0.19	14.30	up
29	13(14)-DiHDPE(A)	DHA	1.17	0.13	47.10	up
30	8-HDHA	DHA	1.26	0.10	2.17	up
31	17(18)-EpETE	EPA	1.31	0.08	6.57	up
32	14(15)-EpETE	EPA	1.24	0.10	46.19	up
33	14(15)-DiHETE	EPA	1.79	0.01	7.35	up
34	5,6-DIHETE	EPA	1.38	0.06	36.58	up
35	9-HEPE	EPA	1.04	0.17	Inf	up
36	17(18)-DiHETE	EPA	1.42	0.13	2.06	up
37	15-HEPE	EPA	1.04	0.17	Inf	up
38	18-HEPE	EPA	1.14	0.13	53.12	up
39	11(12)-DiHETE	EPA	1.10	0.15	Inf	up
40	12(13)-DiHOME	LA	1.13	0.15	65.92	up
41	12,13-EpOME	LA	1.22	0.11	34.49	up
42	9(S),12(S),13(S)-TriHOME	LA	1.20	0.07	2.70	up
43	9,10-EpOME	LA	1.21	0.11	39.56	up
44	9,10-DiHOME	LA	1.15	0.14	54.98	up

Note: ALA, α-linolenic acid; ARA, arachidonic acid; DHA, docosahexaenoic acid; EPA, eicosapentaenoic acid; DGLA, dohomo-γ-linolenic acid; LA, linoleic acid.

## Data Availability

The raw sequence data and oxylipins data reported in this paper have been deposited in the China National Center for Bioinformation/Beijing Institute of Genomics, Chinese Academy of Sciences (GSA: CRA030364, OMIX011987) that are publicly accessible at https://ngdc.cncb.ac.cn/gsa (accessed on 23 September 2025).

## Supplementary Materials

The following supporting information can be downloaded at: https://www.mdpi.com/article/10.3390/ani15192824/s1, Table S1: Ingredients and chemical composition of the diet (air-dry basis). Table S2: The linear equations for the standard curve of oxylipins. Table S3: Information of oxylipins detected between two groups. Table S4: Sequencing data quality of testis between two groups. Table S5: Information of transcriptomic sequencing between two groups.

## Abbreviations

The following abbreviations are used in this manuscript:

ALA	α-Linolenic acid (ALA)
ARA	Arachidonic acid
COX	Cyclooxygenase
Cu/Zn-SOD	Cu,Zn-superoxide dismutase
CYP450	Cytochrome P450
DEGs	Differentially expressed genes
DGLA	Dohomo-γ-linolenic acid
DHA	Docosahexaenoic acid
DHETs	Dihydroxyeicosatrienoic acids
EET	Epoxyeicosatrienoic acid
EPA	Eicosapentaenoic acid
FPKM	Fragments per kilobase of transcript per million mapped reads
GPX3	Glutathione Peroxidase 3
GPX4	Glutathione Peroxidase 4
KEGG	Kyoto encyclopedia of genes and genomes
LA	Linoleic acid
LOX	Lipoxygenase
LXB4	Lipoxin B4
MDA	Malondialdehyde
PCR	Polymerase chain reaction
PG	Prostaglandins
PGD2	Prostaglandin D2
PPARα	Peroxisome proliferator-activated receptor alpha
PUFA	Polyunsaturated fatty acid
qRT-PCR	Quantitative real-time reverse transcription polymerase chain reaction
ROS:	Reactive oxygen species
SOD	Superoxide dismutase
T-AOC	Total antioxidant capacity
TX	Thromboxanes
UPLC–MS/MS	Ultra-performance liquid chromatography tandem mass spectrometry
